# *Pholiota nameko* Polysaccharides Promotes Cell Proliferation and Migration and Reduces ROS Content in H_2_O_2_-Induced L929 Cells

**DOI:** 10.3390/antiox9010065

**Published:** 2020-01-10

**Authors:** Tzu-Jung Sung, Yu-Ying Wang, Kai-Lun Liu, Chun-Hsu Chou, Ping-Shan Lai, Chang-Wei Hsieh

**Affiliations:** 1 Department of Food Science and Biotechnology, National Chung Hsing University, 145 Xingda Rd., South Dist., Taichung City 402, Taiwan; vi840811@gmail.com (T.-J.S.); sicawang1206@gmail.com (Y.-Y.W.); changeonestune@gmail.com (K.-L.L.); 2 Dr. Jou Biotech Co., Ltd., No.21, Lugong S. 2nd Rd., Lukang Township, Changhua Country 505, Taiwan; dr.jason.jou@gmail.com; 3 Department of Chemistry, National Chung Hsing University, 145 Xingda Rd., South Dist., Taichung City 402, Taiwan; pslai@email.nchu.edu.tw; 4 Department of Medical Research, China Medical University Hospital, Taichung 404, Taiwan

**Keywords:** *Pholiota nameko*, antioxidant, reactive oxygen species, scratch assay, proliferation

## Abstract

*Pholiota nameko*, a type of edible and medicinal fungus, is currently grown extensively for food and traditional medicine in China and Japan. It possesses various biological activities, such as anti-inflammatory, anti-hyperlipidemia and antitumor activities. However, *P. nameko* has rarely been discussed in the field of dermatology; identifying its biological activities could be beneficial in development of a new natural ingredient used in wound care. To evaluate its in vitro wound healing activities, the present study assessed the antioxidant and anti-collagenase activities of *P. nameko* polysaccharides (PNPs) prepared through fractional precipitation (40%, 60% and 80% (*v*/*v*)); the assessments were conducted using reducing power, hydroxyl radical scavenging activity, dichloro-dihydro-fluorescein diacetate and collagenase activity assays. The ability of PNPs to facilitate L929 fibroblast cell proliferation and migration was assessed using 3-(4,5-dimethylthiazol-2-yl)-2,5-diphenyltetrazolium bromide (MTT) and scratch assays. The findings indicated that, among all fractions, PNP-80 showed the best antioxidant and anti-collagenase activity, as measured by their reducing power (IC_50_ of PNP-80 was 2.43 ± 0.17 mg/mL), the hydroxyl radical scavenging (IC_50_ of PNP-80 was 2.74 ± 0.11 mg/mL) and collagenase activity assay, and significantly reduced cellular ROS content, compared with that of H_2_O_2_-induced L929 cells. Moreover, PNP-80 significantly promoted L929 fibroblast proliferation and migration, compared with the control group. Overall, we suggested that PNP-80 could be a promising candidate for further evaluation of its potential application on wound healing.

## 1. Introduction

Cellular oxidative stress is related to the overproduction of ROS, such as superoxide anions (O_2_•^−^), hydrogen peroxide (H_2_O_2_), and hydroxyl radicals (•OH), and can occur because of exogenous factors as well as endogenous factors, such as aging, diabetes, obesity and vascular disorders [1,2,3,4,5,6]. These factors give rise to excessive ROS production that leads to oxidative stress, which can negatively affect wound healing, directly and indirectly degrade ECM proteins, weaken dermal fibroblast functions, and cause abnormal inflammation, resulting in non-healing wound [7]. Therefore, reducing oxidative stress is crucial for the functioning of fibroblasts during wound healing [8]. The wound-healing process can be aided by antioxidants from natural sources [9]. Furthermore, polysaccharides have been widely indicated they possess great prospect for used in wound healing; for instance, *Bletilla striata* polysaccharide can promote the proliferation of L929 fibroblasts, scavenge cellular ROS, and downregulate the secretion of pro-inflammatory cytokines [10]. *Lentinus edodes* polysaccharides could be essential in ameliorating oral ulcers because of their high antioxidant activity [11]. Moreover, moisture balance in the skin is critical to wound healing; dry environments can delay the migration of epidermal cells and slowdown autolysis, and a moist surrounding for the wound can prevent secondary infections [12]. Therefore, natural extracts with antioxidant, anti-inflammatory and moisturizing properties are potential candidates for accelerating wound healing. *Pholiota nameko* has received increasing attention in recent years because of its nutritive value and unique taste. An increasing number of studies on the bioactivities of *P. nameko*, such as antitumor, antioxidant, and antihyperlipidemic activities, have generated further interest in nameko mushrooms [13,14]. Nowadays, there are a lot of studies paying attention to *P. nameko* extract and polysaccharides for their inflammatory and antioxidant activity; for example, polysaccharides from *P. nameko* possessed significantly anti-inflammatory activity on egg albumin-induced paw edema in animals [15]. In our previous study, *P. nameko* polysaccharides (PNPs), namely PNP-40, PNP-60, and PNP-80, were separated through fractional precipitation of ethanol (40%, 60%, and 80% (*v*/*v*)), demonstrating that PNPs possessed radical scavenging and Fe^2+^-chelating activities, and protective effect against H_2_O_2_-induced cellular damage. Moreover, PNPs exhibited 27.61–29.56% higher moisture retention activity than did glycerol and PNP-80 showed the greatest moisture retention activity of all fractions [16]. These properties mentioned above suggest that PNPs might have potential application for wound healing.

The process of wound healing can be divided into the following phases: inflammatory, proliferative, and remodeling. Several factors could affect the wound-healing process, such as oxidative damage, infection, and venous sufficiency [17]. The migration of fibroblasts to the wound site and their proliferation are particularly essential for wound healing because of their ability to break down the fibrin clot, deposit extracellular matrix (ECM) components and collagen, form granulation tissue and contract the lesion’s borders toward the center in the wound area—all important to the wound-healing process [7]. Furthermore, they secrete various signaling molecules that stimulate macrophage activation, such as interferons (INF)-α, β, and γ, and facilitate keratinocyte differentiation, proliferation, and migration, such as keratinocyte growth factor [18,19]. Accordingly, facilitating the migration and proliferation of fibroblast could be one of the means of improving wound healing.

In addition, chronic wound healing is characterized by the excessive expression of pro-inflammatory cytokines and proteases, such as collagenase and elastase, released from neutrophils in the inflammatory stage, which disrupt the balance between extracellular matrix (ECM) degradation and deposition [20]. Elevated expression of metalloproteinases (MMPs) might break the balance of the healing process in the inflammatory phase [21]. It has been indicated that the topical application of metalloproteinase inhibitors can improve chronic wound [22,23]. Thereby, natural products with inhibition activity against metalloproteinase might imply that they could improve non-healing wounds.

However, PNPs have rarely been discussed in the field of dermatology and the in vitro wound-healing effects of PNPs have yet to be reported. Therefore, the purpose of the present study was to further evaluate the potential application of PNPs on wound healing.

## 2. Materials and Methods

### 2.1. Materials and L929 Cell Line

*P. nameko* cultivated in Puli Township, Nantou County, Taiwan (R.O.C.) was purchased from the Rich Year Farm in Nantou, Taiwan. The mouse fibroblast cell line L929 (ATCC^®^ CCL-1™) sourced from ATCC (Manassas, VA, USA) was cultured in Dulbecco’s modified Eagle’s medium (DMEM, Gibco^®^, Grand Island, NY, USA) containing 10% fetal bovine serum (FBS, Gibco^®^, Grand Island, NY, USA) and antibiotics (100 U penicillin and 100 U/mL streptomycin, Gibco^®^, Grand Island, NY, USA) under 5% CO_2_ at 37 °C. Cells were harvested after reaching confluence by using 0.05% trypsin–EDTA (Gibco^®^, Grand Island, NY, USA). Fresh culture medium was added to produce single-cell suspensions for further incubation. Potassium ferricyanide (C_6_N_6_FeK_3_) and ferric chloride (FeCl_3_) were purchased from Showa Chemical Industry Co., Ltd., Tokyo, Japan. Trichloroacetic acid was purchased from Alfa Aesar, Tewksbury, MA, USA. Sodium salicylate, ferrous sulfate heptahydrate (FeSO_4_), hydrogen peroxide (H_2_O_2_), the fluorescein-labeled dye 2′,7′-dichlorofluorescein diacetate (DCF-DA), 3-(4,5-dimethylthiazol-2-yl)-2,5-diphenyltetrazolium bromide (MTT), and ascorbic acid (vitamin C) were purchased from Sigma-Aldrich, St. Louis, MO, USA. Collagenase from *Clostridium hislolyticum* (Type I, 0.25–1.0 units/mg, Sigma, St. Louis, MO, USA) was purchased from Sigma-Aldrich, USA. All other chemicals used were of analytical grade.

### 2.2. Extraction and Fractionation

PNPs were prepared as described in our previous study [16]. PNPs were extracted using ethanol precipitation. Ethanol was added at final concentrations of 40%, 60%, and 80%, and the resulting PNPs were named PNP-40, PNP-60, and PNP-80, respectively. The three different ethanol precipitations of PNP samples were extracted, lyophilized, and refrigerated at 4 °C.

### 2.3. Reducing Power Assay

The reducing power assay was performed as previously described but with modification [24]. Equal volumes (312.5 µL) of PNPs dissolved in ddH_2_O (0.3125–5.000 mg/mL), phosphate buffer (0.2 M, pH 6.6), and 1% potassium ferricyanide were mixed. The mixture was heated to 50 °C for 20 min; subsequently, 312.5 µL of 10% trichloroacetic acid was added to the mixture, followed by 312.5 µL of distilled water and 62.5 µL of 0.1% ferric chloride. Absorbance was immediately detected at 700 nm. Vitamin C (0.0063–0.1014 mg/mL) was used as the positive control. The IC_50_ value was equal to the concentration of samples producing 0.5 absorbance at 700 nm.

### 2.4. Hydroxyl Radical Scavenging Activity

Hydroxyl radical scavenging activity was assessed using a previously described Fenton reaction, with modification [25]. Briefly, 50 μL of PNPs dissolved in ddH_2_O (0.3125–5.000 mg/mL) was incubated with 50 μL of sodium salicylate (9 mM), 50 μL of FeSO_4_ (9 mM), and 50 μL of H_2_O_2_ (0.025%, *w*/*v*) at 37 °C for 30 min. Absorbance was then determined at 510 nm. Deionized water was used as the blank control, and vitamin C (0.3125–5.000 mg/mL) was served as the positive control. Hydroxyl radical scavenging activity was calculated using the following equation:


Hydroxyl radical scavenging activity (%) = (absorbance of blank control − absorbance of sample/absorbance of blank control) × 100%

IC_50_ of PNPs was derived from the formula, Y = 100 × A1/(A1 + X), using GraphPad Prism 6.01 and Y denotes the relative content of hydroxyl radical (Y = 100 when X = 0), A1 denotes IC_50_ of PNPs and X denotes the concentration of PNPs [26].

### 2.5. Measurement of Inhibitory Effect on Collagenase

Inhibitory effect on collagenase was performed by modified Wang’s method [27]. To measure the collagenase activity, 100 μL of 200 units/mL collagenase and 100 μL PNPs (5000, 2500, 1250 μg/mL) were mixed together and incubated at 37 °C for 15 min, weighed at 1 mg of azo dye-impregnated collagen substrate and mixed with 800 μL 0.1 M Tris-HCl buffer (pH 7.0), then added together at 43 °C for 1 h under shaking conditions. Subsequently, the reaction mixture was centrifuged at 3000 rpm for 10 min, and the absorbance was read at 520 nm using the ELISA reader. Distilled water was used as control, and 100 μL of epigallocatechin gallate (EGCG, 5000, 2500, 1250 μg/mL) was used as positive control.

The inhibitory rate of collagenase (%) = (A_c_ − A_ts_/A_c_) × 100%

where A_c_ represents the absorbance of the control, and A_ts_ represents the absorbance of the test sample.

### 2.6. ROS Generation

The ROS generation assay was performed using a previously described method, with modification [28]. Specifically, the concentration of ROS was evaluated using a DCF-DA probe (Sigma Aldrich). The L929 cells were seeded in 24-well plates at a concentration of 1.5 × 10^4^ cells/well and allowed to adhere for 24 h. The cells were pre-incubated with PNPs (500 μg/mL) for 24 h and then exposed to H_2_O_2_ (0.75 mmol/L) for an additional 2 h after adhesion. After treatment, the L929 cells were incubated in DMEM, without FBS, containing DCF-DA (10 μM) at 37 °C in the dark for 30 min. After removal of the probe by washing twice in phosphate-buffered saline (PBS), the final results were evaluated using fluorescence microscopy (Olympus IX51, Tokyo, Japan). Images were captured, and the mean density values were analyzed using Image J software.

### 2.7. Cell Proliferation

Cell proliferation was determined through the MTT assay by using a previously described procedure, with modification [29]. Briefly, L926 cells were seeded in three 96-well plates (1800 cells/well). After 24 h (day 0), the PNPs (500 µg/mL) dissolved in DMED medium were added and then incubated for 0 h, 24 h and 48 h at 37 °C with 5% CO_2_ in a humidified atmosphere, followed by 100 µL of MTT, and the plate was incubated for 2 h. Absorbance was measured at a test wavelength of 570 nm to evaluate cell proliferation. Absorbance was measured on days 0, 1, and 2.

### 2.8. Scratch Assay

The scratch assay—typically used to evaluate the wound-healing capacity of a substance or molecule—was performed using a previously described method, with modification [30]. The assay was used to study cell migration and proliferation, which are crucial for tissue repair. L929 cells were seeded (10^5^ cells/mL) in a 24-well plate and cultured for 24 h. Cell culture monolayers were scratched with a sterile 200-μL pipette tip across the center of the well. After scratching, the wells were gently washed twice with PBS to remove the detached cells. The cells were then treated with 500 μg/mL of the three different PNPs prepared in DMED medium. Control cells were not treated with any PNP. Wound-healing efficiency was monitored at 0 and 24 h. The scratch closure rate is expressed as the percentage of scratch closure on an initial area basis, according to the following Equation:

Scratch closure rate = (A_t0_ − A_t_/A_t0_) × 100,

where A_t0_ is the scratch area at time 0 h and A_t_ is the corresponding scratch area at 24 h. The values shown are the means of three wells from three independent experiments.

### 2.9. Microscopy and Image Analysis

Scratch wound closure was examined using an inverted microscope, and images were captured and analyzed using fluorescence microscopy (Olympus IX51) software. The scratch closure area was monitored at different time intervals (0 and 24 h) to calculate wound closure [31].

### 2.10. Statistical Analysis

All data are expressed as means ± standard deviations. Statistical data processing was implemented through dispersion analysis using SPSS 20 software. Statistical analysis was performed using one-way ANOVA and Duncan’s multiple range tests, and a *p* value of < 0.05 was considered to indicate statistical significance.

## 3. Results and Discussion

### 3.1. Reducing Power and Hydroxyl Radical Scavenging Activity

**Figure 1:**
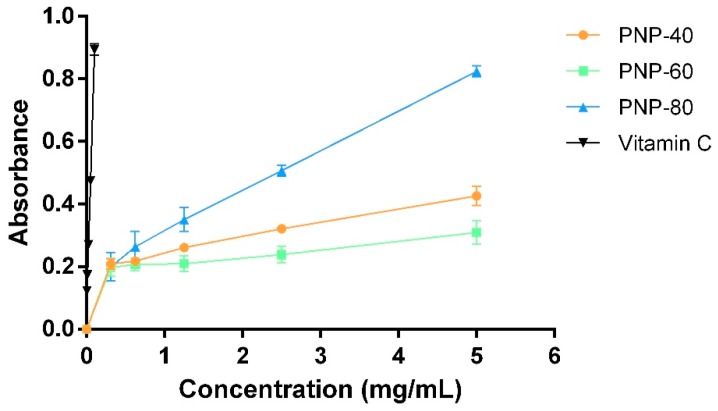
Reducing power of PNPs and vitamin C at different concentrations. The experiments were conducted in triplicate independently (*n* = 3), and the data are expressed as the means ± standard error (SE).

It was indicated that the antioxidant activities of natural compounds are associated with their wound-healing properties [9]. Therefore, to evaluate the antioxidant activity of PNPs, we used two different antioxidant assays, the reducing power and hydroxyl radical scavenging activity, to simulate the environmental oxidative stress in non-healing wound. Hydroxyl radicals overproduced via uncontrolled Fenton reaction disturb healing process in delayed wound healing [32]. The reducing power can be used to assess to extent to which a compound reduces Fe^3+^ to Fe^2+^, and the higher absorbance value at 700 nm is, the stronger is antioxidant [33]. The reducing power levels derived from PNPs and vitamin C increased in a dose-dependent manner in Figure 1. PNP-80 (IC_50_ = 2.43 ± 0.17 mg/mL) had a higher reducing power for ferric ion than did PNP-40 and PNP-60 (*p* < 0.05). The growth rates of PNP-40 and PNP-60 were slow, with the absorbance values being 0.43 and 0.31 at 5.0 mg/mL, respectively.

**Figure 2:**
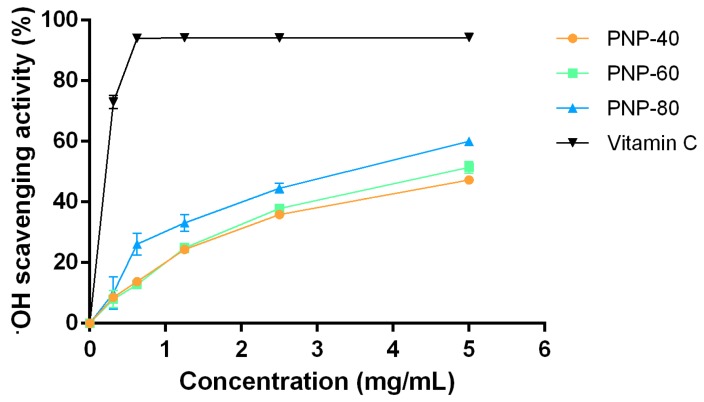
Hydroxyl radical scavenging activity of PNPs and vitamin C. The experiments were conducted in triplicate independently (*n* = 3), and the data are expressed as the means ± standard error (SE).

Hydroxyl radicals are among the most reactive and hazardous free radicals. Overproduction hydroxyl radical overwhelms oxidation-reduction system and causes damage in cellular protein, DNA, lipid and wound-healing related cells, fibroblast, arising delayed healing of wounds [9]. Therefore, to evaluate the hydroxyl radical scavenging activity of the PNPs in vitro, we used the Fenton reaction system as a model. The hydroxyl radical scavenging activities of the PNPs and vitamin C are illustrated in Figure 2. All samples exhibited hydroxyl radical scavenging activity in a dose-dependent manner. The IC_50_ value of PNP-80 and PNP-60 was 2.74 ± 0.11 mg/mL (95% confidence intervals = 2.520 to 2.965 mg/mL and R^2^ = 0.9406) and 4.25 ± 0.09 mg/mL (95% confidence intervals = 4.069 to 4.436 mg/mL and R^2^ = 0.9816), respectively; additionally, at 5 mg/mL, the scavenging abilities of PNP-80, PNP-60, PNP-40, and vitamin C were 60.17%, 51.44%, 47.25%, and 94.29%, respectively. These results indicate that PNP-80 exhibited greater potency to donate hydrogen to hydroxyl radicals than did the other fractions (*p* < 0.05) [34].

Low-molecular-weight polysaccharides have a less compact structure than do high-molecular-weight polysaccharides. This signifies that low-molecular-weight polysaccharides have more free functional groups, such as carboxyl, amino, and hydroxyl groups which could react with free radicals and then stabilize them, than do high-molecular-weight polysaccharides [35]. The antioxidant properties of chitosan weighing 2.2–300.0 kDa are inversely related to their molecular weights [36], explaining why PNP-80 (4.40 kDa) exhibited a more pronounced scavenging activity (measured through its reducing power and hydroxyl radical scavenging activity) than did PNP-60 (21.57 kDa) and PNP-40 (333.49 kDa) at a concentration of 5 mg/mL. In terms of reducing power, the absorbance value at 700 nm of *G. lucidum* polysaccharides-80 was 0.138 at a concentration of 2.0 mg/mL [37], suggesting that PNP-80 could be more effective electron donors that can react with free radicals and convert them into more stable products. In terms of hydroxyl radical scavenging activity, the IC_50_ value of *Auricularia auricular* polysaccharides that was reported to be greater than 5.0 mg/mL is similar to that of PNPs [38]. Our results show that among the three fractions, PNP-80 had the greatest antioxidant activity. Moreover, although PNPs did not exhibit antioxidant activity as good as vitamin C did, PNPs possess a variety of wound healing-related functions, such as moisturizing [16] which vitamin did not hold.

### 3.2. Anti-Collagenase Activity of PNPs

**Figure 3:**
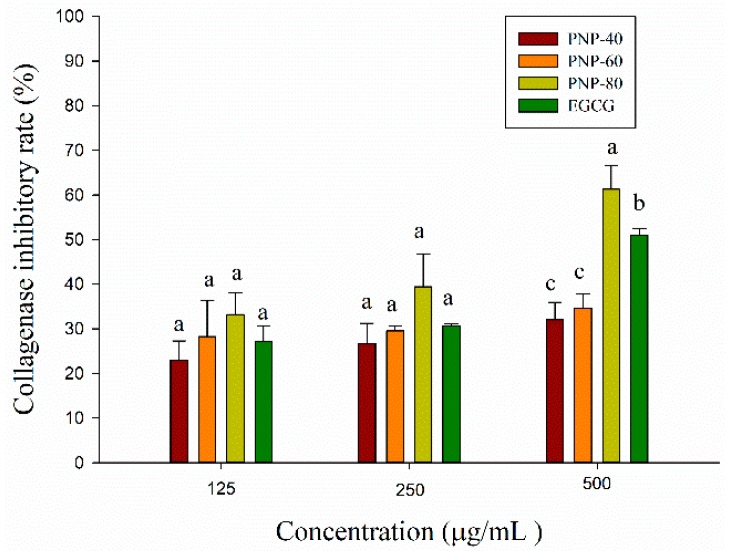
Anti-collagenase activity of PNPs (125, 250, 500 μg/mL) and EGCG (125, 250, 500 μg/mL). The experiments were conducted in triplicate independently (*n* = 3), and the data are expressed as the means ± standard error (SE). ^a–c^ Means within the same concentration followed by the same letter were not significantly different (*p* > 0.05).

Collagenase is one of matrix metalloproteinases (MMPs) capable of breaking down the extracellular matrix (ECM), the major components of connective tissue, which can support the skin structure, maintain skin elasticity and play an important role in wound healing [39]. However, it has been shown that overexpression of collagenase was associated with chronic wound and treatment chronic wound with metalloproteinase inhibitor could improve delayed healing wound [22,23]. To evaluate if PNPs have the inhibitory ability against collagenase, we used in vitro collagenase activity assay as an evaluation model. Epigallocatechin gallate (EGCG), the predominant catechin in tea, possesses the inhibition of collagenolytic activity by collagenase and is usually used as positive control in collagenase activity assay [40,41,42]. The collagenase inhibitory activity of PNPs and epigallocatechin gallate (EGCG) was shown in Figure 3. All of samples inhibited collagenase in a dose-dependent manner at a range of concentration from 125 to 500 μg/mL. The inhibitory activity of PNP-40, PNP-60 and PNP-80 ranged from 25% to 33%, from 26% to 39% and from 32% to 61% at 125–500 μg/mL, respectively. The inhibitory activity of PNP-80 at a concentration of 500 μg/mL was significantly higher than that of PNP-60, PNP-40 and EGCG. Although the collagenase inhibitory activity of all samples did not show significant differences at a concentration of 125 μg/mL and 250 μg/mL, the inhibitory activity of PNP-80 was slightly higher than that of PNP-40, PNP-60, and EGCG. The result indicated that PNPs might improve delayed healing wound by reducing the collagenase activity.

Collagenases are a group of zinc-containing proteinases, which contains Zn ion at its active site that facilitates interaction with an inhibitor [43]. Previous studies showed that PNPs have the ability to chelate metal [16], which might suggest that the inhibitory mechanism of PNPs against collagenase is due to their ability to chelate Zn ion at collagenase’s active site and hamper the interaction between the substrate and its active site.

### 3.3. Effect of Intracellular ROS Generation

**Figure 4:**
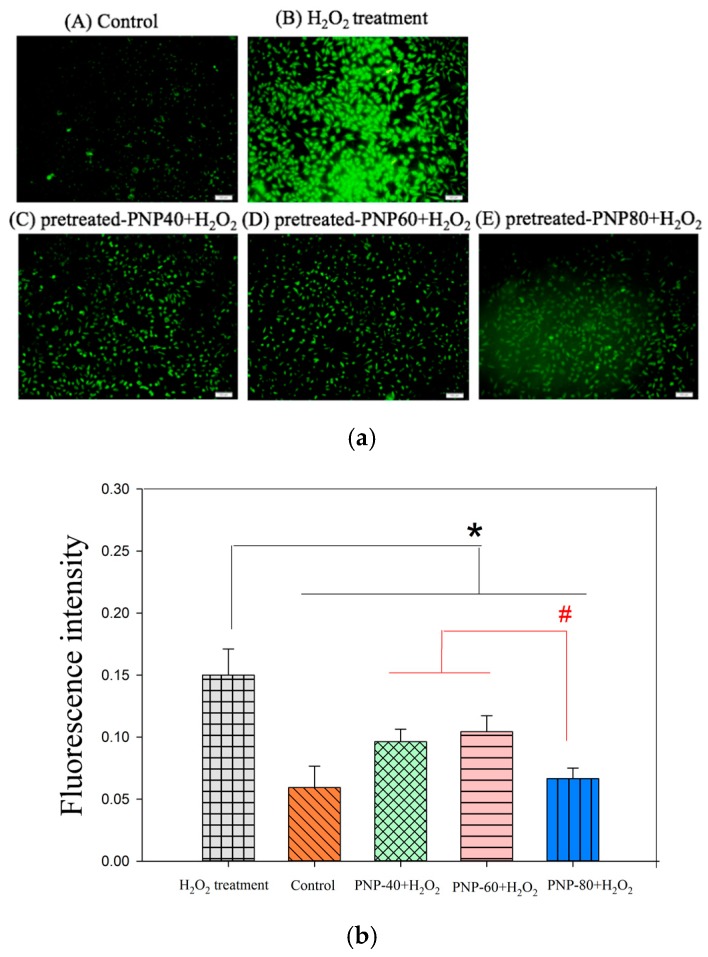
(**a**) Radical scavenging activity of PNPs (500 µg/mL) on L929 cells against H_2_O_2_-induced ROS generation (scale bar = 100 μm). Control (A), H_2_O_2_-induced L929 cells without PNPs pre-treatment (B), with PNP-40 pre-treatment (C), with PNP-60 pre-treatment (D), and with PNP-80 pre-treatment (E). (**b**) Quantitative analysis of the radical scavenging effect of PNPs-pretreated on L929 cells against H_2_O_2_-induced ROS generation. The experiments were conducted in triplicate independently (*n* = 3), and the data are expressed as the means ± standard error (SE) (* *p* < 0.05 compared with H_2_O_2_-treated L929 cells without PNPs; # *p* < 0.05 compared with PNPs-pretreated against H_2_O_2_-induced L929 cells).

Wound healing is a dynamic and precisely controlled process and can be divided into three phases: inflammatory, proliferative and remodeling phase. During the inflammatory phase, neutrophils infiltrate to the wound area in order to combat microbes and clear cell debris via secreting H_2_O_2_ and proteases. Even though these substances have a beneficial effect on the wound healing process, overproduction H_2_O_2_ gives rise to the non-healing wound via weakening wound-healing related cell, fibroblast [17,44]. In our previous study, we proved that pre-treated with PNPs reduced the H_2_O_2_-induced damage to L929 cell using MTT assay, suggesting PNPs might protect L929 cell from programmed cell death by ameliorating oxidative stress in L929 cell [16]. To assess if PNPs possess the ability against H_2_O_2-_induced oxidative stress, we utilized ROS generation assay as a model. We had already proved that PNPs did not exhibit cytotoxicity toward L929 cells under a concentration of 500 µg/mL [16]. The levels of ROS, determined through DCFH-DA staining, produced in control cells, H_2_O_2_-induced cells without PNPs pretreatment and with PNPs pretreatment (500 µg/mL) are shown in Figure 4a; in this figure, green fluorescence indicates ROS production. This study observed a notable increase in ROS production in the H_2_O_2_-induced cells without PNPs, however, the ROS levels were lower in the PNPs-pretreated groups. ROS production was also quantified using image J software (Figure 4b). Compared with the H_2_O_2_-induced L929 cells without PNPs pre-treatment, the H_2_O_2_-induced L929 cells pre-treated with PNPs had significantly reduced ROS levels. The relative fluorescence intensity of H_2_O_2_-induced L929 cells without PNPs was 0.15, H_2_O_2_-induced L929 cells pre-treated with PNP-80, PNP-60 and PNP-40 which were reduced to 0.06, 0.10, and 0.09, respectively. Overall, PNP-80 showed the greatest free-radical scavenging activity and reduced the level of ROS in the H_2_O_2_-treated cells by 53.33%. Moreover, it was indicated *P. nameko* polysaccharides have the potential to increase the activity of cellular antioxidant enzymes, such as superoxide dismutase (SOD), thereby reducing cellular ROS contents [45], suggesting PNP-40, PNP-60 and PNP-80 might also ameliorate cellular oxidative stress via up-regulating SOD expression. According to these results, we infer that PNPs could attenuate H_2_O_2_-induced damage via improving cellular oxidative stress. These results are similar to that of sulfated polysaccharides isolated from the edible marine algae *Padina tetrastromatica* which weaken H_2_O_2_-induced cellular damage via the reduction of intracellular reactive oxygen species level [46].

### 3.4. Cell Proliferation and Migration

Proliferation and recruitment of fibroblasts in the wound area are particularly important to wound healing process because fibroblasts are directly liable for depositing ECM, forming granulation tissues and contracting wound lesions. Consequently, substances that could enhance the proliferation and migration activity of fibroblast implies they have the potential to accelerate the wound healing process [47].

**Figure 5:**
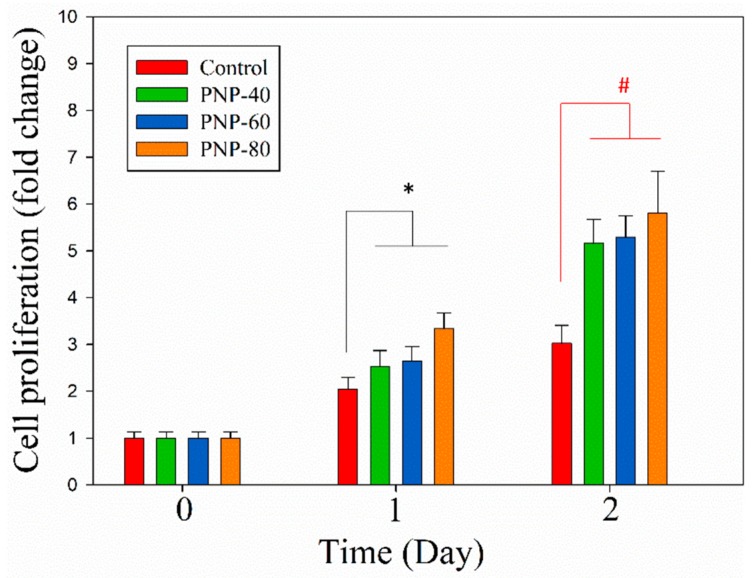
Proliferation of L929 cells treated with PNPs (500 µg/mL) for 0, 1, and 2 days (relative to day 0). The experiments were conducted in triplicate independently (*n* = 3), and the data are expressed as the means ± standard error (SE) (* *p* < 0.05 compared with control cells on day 1; # *p* < 0.05 compared with control cells on day 2).

In wound healing, a major concern is the positive response of fibroblasts toward the materials of interest; L929 is widely used for testing if the samples possess the stimulation activity by studying its cell proliferation activity [48,49]. The MTT assay was used to determine the proliferation of L929 fibroblast cells. Proliferative activity can be determined by measuring the reduction of yellow tetrazolium salt to purple formazan crystals, which indicates cells’ metabolic state [50]. The relative fold changes in cell proliferation are shown in Figure 5. The proliferative activity of L929 cells treated with PNP-80 increased significantly compared with that of the control cells on day 1. However, on day 2, all three PNPs significantly promoted the proliferative activity of L929 cells compared with that of the control cells (*p* < 0.05). The proliferative activity of L929 cells treated with PNP-80, PNP-60, and PNP-40 exhibited 5.80-, 5.29-, and 5.16-fold changes on day 2 compared with the changes observed on day 0, respectively, which were significantly higher than those in the control cells. These results indicate that in L929 cells, PNPs greatly promote proliferation, which is crucial in skin wound healing.

**Figure 6:**
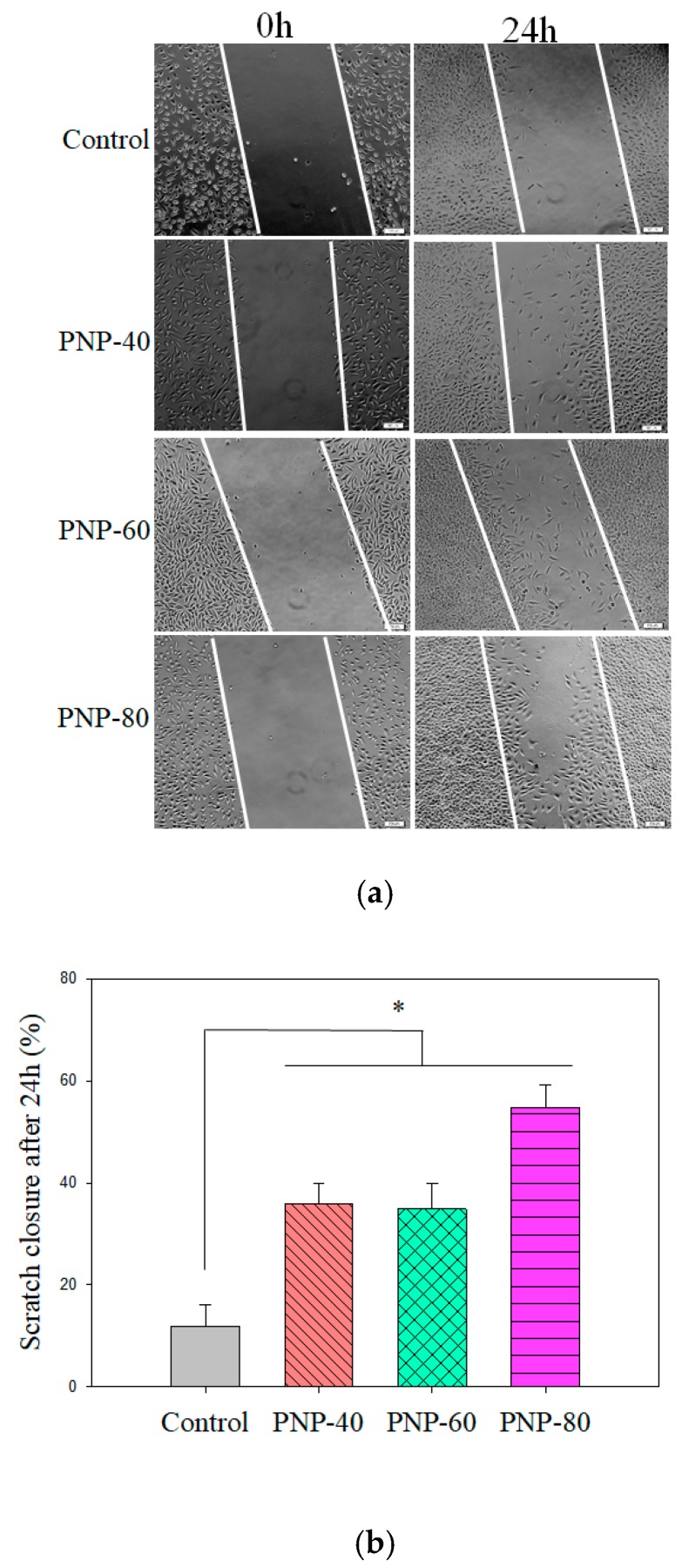
(**a**) L929 fibroblast cells treated PNPs (500 µg/mL) and control group observed after injury to the cell monolayer by using an in vitro scratch assay immediately after scratching (0 h) and 24 h after scratching (scale bar = 100 μm). (**b**) Quantitative analysis of L929 fibroblast cell scratch closure 24 h after the scratch assay. The experiments were conducted in triplicate independently (*n* = 3), and the data are expressed as the means ± standard error (SE) (* *p* < 0.05 compared with control cells; # *p* < 0.05 compared with PNP-treated cells).

We next determined whether PNPs affect L929 cell migration. Cellular migration is an essential process in wound and cutaneous repair, and fibroblasts can traverse tissue environments to degrade, repair, and remodel the ECM [51]. We used the in vitro scratch assay to measure the migration of L929 cells into a cell-free gap in the tissue culture dish. As presented in Figure 6a,b, during the time from 0 to 24 h, PNPs significantly increased the closure speed in the cells relative to the control cells (11.83%). L929 cells that were treated with PNPs showed a significant increase in closure rate, with the rates being 35.82% (PNP-40), 34.81% (PNP-60), and 54.75% (PNP-80). The closure rates of L929 cells treated with PNPs at 24 h were higher than those of the control cells by 194.25–362.80%. The increased migratory activity of L929 cells treated with PNPs suggests that PNPs have the potential to enhance cutaneous repair [47,52].

In human skin fibroblasts, 24-h exposure to ammonium–chitosan conjugates was reported to result in a significantly increased closure rate (approximately 70%) compared with the rate observed in control cells (approximately 45%) [53]. *Sargassum ilicifolium* aqueous extracts were also reported to engender an enhanced wound closure rate (97.83%) in L929 cells at 24 h compared with the rate observed in control cells (46.11%); hence, the wound closure rate of the treated cells was higher than that of the control cells by 112.17% [31], indicating PNPs demonstrated better enhancement in fibroblast migration.

It was indicated that β-glucans are a multi-functional modulator of wound healing [54], for instance, (1→3)-(1→6)-β-d-glucan from *Aureobasidium pullulans* stimulates dermal fibroblast proliferation and migration [55]. Moreover, our previous study showed that the β-d-glucan contents in PNP-40, PNP-60 and PNP-80 were 20.20%, 12.20% and 10.15%, respectively [16], and other report was indicated as a β-d-glucan-(1→3)-linked, substituted at *O*-6 by β-d-Glcp or (1→6)-linked β-d-Glcp side chains using NMR and methylation analyses [56], suggesting that β-d-glucan might be one of active substances in PNPs elevating cell migration and proliferation.

A moist wound environment has a variety of beneficial effects on wound healing process, such as prevention of tissue dehydration and cell death, accelerated angiogenesis, increased breakdown of dead tissue and fibrin, compared with a dry wound environment [57]. Our previous study showed the moisture-retention rate of PNP-80 was 64.17% after 96 h exposed to 10% relative humidity (RH) and it was higher than that of PNP-40 (63.42%) and PNP-60 (63.21%) and far higher than that of glycerol (49.53%) [16]. In this study, we found that PNP-80 exhibited the best reducing power and hydroxyl radical scavenging activity of all fractions, greatly reduced ROS content in H_2_O_2_-induced L929 cells and significantly enhanced the proliferation and migration rate of L929 cells, compared with control group, suggesting that PNP-80 might be a promising candidate for further evaluation of its potential application on wound healing. In addition, we did further assess if PNPs have the antibacterial activity against *Escherichia coli* and *Staphylococcus aureus* (the data was not shown here). Although the result indicated that PNPs did not exhibit antibacterial activity against *E. coli* and *S. aureus* in agar well diffusion assay at a concentration of 10 mg/mL, it does not mean PNPs are not appropriate to be used as functional ingredients in wound healing related agents. That is because the flaw might be made up by adding additional antibacterial agents.

Furthermore, identifying and refining the active compounds in PNPs using gel filtration chromatography and ion-exchange liquid chromatography is important and necessary because PNPs did not show great potency in in vitro wound healing assay.

## 4. Conclusions

In our study, PNPs extracted using fractional precipitation show great antioxidant activity in vitro; additionally, they significantly reduce ROS production, promote proliferation, and increase the wound closure rate at the cellular level. These attributes provide information for the first time on the effectiveness of PNPs in enhancing in vitro wound healing. The findings indicate that PNP-80 shows the greatest antioxidant, anti-collagenase, proliferative, and migratory activities among the fractions. PNP-80 was the most promising candidate of all fractions for further evaluation of its potential application on wound healing. Further investigations into the effects of PNPs on wound healing must be conducted on an animal model and their stability; moreover, the structure and mechanisms underlying the wound-healing effects of PNPs warrant investigation.
